# GABA_A_ receptor dysfunction in autism spectrum disorder: molecular mechanisms and therapeutic opportunities

**DOI:** 10.3389/fphar.2026.1840553

**Published:** 2026-06-08

**Authors:** Durairaj Ragu Varman, Bhagavathi Sundaram Sivamaruthi, Periyanaina Kesika, Ataúlfo Martínez-Torres, Daniel Reyes-Haro, Chaiyavat Chaiyasut

**Affiliations:** 1 School of Biomedical Sciences, Sri Balaji Vidyapeeth (Deemed-to-be-University), Puducherry, India; 2 Office of Research Administration, Chiang Mai University, Chiang Mai, Thailand; 3 Innovation Centre for Holistic Health, Nutraceuticals, and Cosmeceuticals, Faculty of Pharmacy, Chiang Mai University, Chiang Mai, Thailand; 4 Departamento de Neurobiología Celular y Molecular, Laboratorio de Neurobiología Molecular y Celular, Instituto de Neurobiología, Universidad Nacional Autónoma de México, Mexico City, Mexico

**Keywords:** autism spectrum disorder, excitation-inhibition imbalance, GABA_A_ receptors, interneuron dysfunction, subtype-selective positive allosteric modulators

## Abstract

Autism spectrum disorder (ASD) comprises diverse neurodevelopmental trajectories in which altered circuit dynamics converge on a disturbance of excitation–inhibition balance. Genetic, *postmortem*, neuroimaging, and model-system evidence implicates γ-aminobutyric acid type A (GABA_A_) receptors as a major molecular node within this imbalance. ASD has been associated with changes in GABA_A_ receptor subunit expression and assembly, notably within 15q11-q13 clusters, impaired receptor trafficking and synaptic anchoring, and a shift in the relative contribution of phasic (synaptic, containing the γ2 subunit) *versus* tonic (extra-synaptic with δ or ρ) inhibition. *Postmortem* studies reported reduced expression of GABA_A_ subunits, which correlates with decreased expression of glutamic acid decarboxylase 65/67, interneuron loss, particularly in parvalbumin networks that support gamma oscillations, and disrupted chloride homeostasis, which can delay the developmental “GABA switch” from depolarizing to hyperpolarizing signalling. Here, we review mechanistic advances across environmental and monogenic ASD models [e.g., prenatal valproate exposure, maternal immune activation, *shank3* (SH3 and multiple ankyrin repeat domains 3), *fmr1* (fragile X messenger ribonucleoprotein 1), and *mecp2* (methyl-CpG binding protein 2) alongside human biomarker studies using proton magnetic resonance spectroscopy and positron emission tomography, highlighting how GABA receptor subtype- and circuit-specific inhibitory deficits map onto sensory, social, cognitive, and comorbid seizure phenotypes. We evaluate emerging therapeutic strategies that move beyond nonselective sedation, including α2/α3-and α5-preferring positive allosteric modulators, neurosteroid-based enhancers of tonic inhibition, and interventions targeting sodium-potassium chloride cotransporter 1/potassium chloride cotransporter 2-regulated chloride gradients, as well as activity-dependent approaches such as environmental enrichment. Finally, we outline priorities for precision translation: multimodal biomarker-guided stratification, developmentally timed intervention windows, and trials aligned to receptor subtype pharmacology and circuit endpoints. This review integrates molecular and translational research on GABA_A_ receptor dysfunction in ASD, emphasizing key mechanistic insights and potential therapeutic approaches. To develop precise GABAergic treatments, a comprehensive strategy that includes molecular profiling, biomarker-guided clinical trials, and insights from developmental neuroscience is necessary.

## Graphical Abstract

**Figure:**
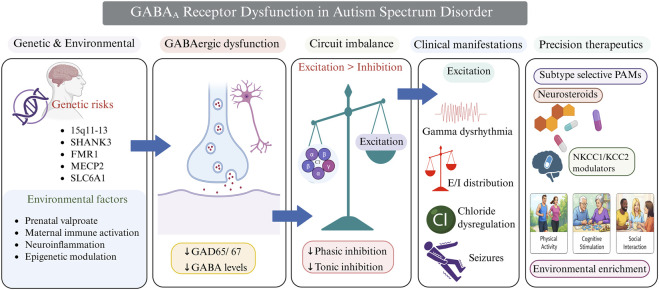
GABA_A_ receptor dysregulation in ASD arises from genetic factors, including 15q11-q13 GABA receptor clusters, *shank3*, *fmr1*, *mecp2*, and SLC6A1, together with environmental influences that disrupt GABAergic development. Reduced GAD65/67 and altered GABA levels impair phasic and tonic inhibition, leading to E/I imbalance, gamma rhythm abnormalities, chloride dysregulation, seizures, and network instability. Emerging personalized therapies target GABA_A_ receptor subtypes, neurosteroids, chloride transporters, and environmental modulation to restore inhibitory balance.

## 1 Introduction

Autism Spectrum Disorder (ASD) is a heterogeneous neurodevelopmental condition characterized by persistent impairments in social communication and interaction, accompanied by restricted interests and repetitive patterns of behavior. The disorder emerges early in development and persists across the lifespan, frequently accompanied by intellectual disability, epilepsy, anxiety disorders, and sensory processing abnormalities. Epidemiological studies indicate a rising global prevalence, now estimated at approximately 1%–2% of the population, with a consistent male predominance (Jiang et al., 2022; Al Dera, 2022). The clinical and genetic diversity observed in individuals with ASD points to a complex, multifactorial origin, shaped by dynamic interactions among genetic predispositions, epigenetic modifications, and environmental influences (Lord et al., 2018; Grove et al., 2019). Although improvements in diagnostic criteria and awareness have contributed to increased identification rates, converging evidence supports a substantial biological and genetic basis for ASD (Marilovtseva et al., 2025).

Over the last 20 years, advances in genomic technologies such as whole-exome and whole-genome sequencing have shown that ASD is linked to rare *de novo* mutations, copy number variations, and polygenic risk factors. These genes play roles in synaptic development, chromatin remodeling, transcription, and intracellular signaling (Jiang et al., 2022; Al Dera, 2022). Although there is genetic variability, many ASD-associated genes tend to impact common neurobiological pathways, especially those related to synaptic function and the balance of excitatory and inhibitory signals (Braat and Kooy, 2015; Gordon et al., 2026). This overlap supports the idea that ASD partly stems from neural circuit dysregulation. One key neurobiological theory highlights the importance of the excitatory–inhibitory (E/I) balance. In the early 2000s, it was proposed that essential cognitive and social processes depend on the precise regulation between glutamatergic excitatory neurons and GABAergic inhibitory interneurons (Rubenstein and Merzenich, 2003). When this balance is disrupted, either by excessive excitation or diminished inhibition, it can destabilize cortical microcircuits, disrupt oscillatory synchronization, and impair information processing (Marilovtseva et al., 2025). Consistent findings from *postmortem* studies and animal models of ASD show decreased glutamic acid decarboxylase (GAD65/67) expression, changes in Gamma-aminobutyric acid (GABA) receptor subunit composition, and deficits in specific interneuron populations, including parvalbumin-positive basket cells (Marilovtseva et al., 2025; Al Dera, 2022).

GABA is the main inhibitory neurotransmitter in the mammalian central nervous system. It plays a crucial role in controlling neuronal excitability, neural plasticity during key developmental stages, and the formation of neural circuits (Ben-Ari, 2002; Cellot and Cherubini, 2014). During early neurodevelopment, GABA initially exerts a depolarizing effect because of elevated intracellular chloride concentrations maintained by Na-K-2Cl cotransporter isoform 1(NKCC1). As development continues, GABA transitions to an inhibitory role (hyperpolarization) as K-Cl cotransporter isoform 2 (KCC2) expression increases (Kaila et al., 2014). Aberrant shifts from excitatory to inhibitory GABAergic signaling are frequently reported in both preclinical and clinical ASD studies (Tyzio et al., 2014; Ben-Ari, 2014). E/I imbalance is particularly notable in cortical and limbic regions, which are crucial for social behaviors and sensorimotor processes (Nelson and Valakh, 2015; Sohal and Rubenstein, 2019). Within this framework, heightened glutamatergic excitation or reduced GABAergic inhibition disrupts synaptic development and network stability, contributing to core ASD features including sensory hypersensitivity, impaired social interaction, and reduced cognitive flexibility (Nelson and Valakh, 2015; Borghese et al., 2025; Kang and Delahhantry et al., 2026; Alruwaili et al., 2026).

GABAergic neurotransmission involves ionotropic GABA_A_ and metabotropic GABA_B_ receptors. GABA_A_ receptors function as ligand-activated chloride channels, enabling rapid synaptic inhibition, and are some of the most chemically diverse receptor types in the brain. These heteropentameric complexes result in considerable heterogeneity based on brain region, cell type, and subcellular location (Ghit et al., 2021; Marilovtseva et al., 2025). Receptors with α1–3βγ subunits at synapses mainly govern phasic inhibition, whereas extrasynaptic δ-containing receptors play a role in tonic inhibitory currents (Ghit et al., 2021). Multiple lines of evidence suggest that GABA_A_ receptor dysfunction is a common feature across ASD models. Genetic research has identified polymorphisms and copy number variations in GABA_A_ receptor subunit genes, especially on chromosome 15q11–q13, that are associated with an increased risk of autism (Braat and Kooy, 2015; Al Dera, 2022). *Postmortem* analyses reveal altered levels of α5, β3, and γ2 subunits in the cortical and hippocampal regions, which are critical for social cognition (Marilovtseva et al., 2025). Furthermore, experimental models such as prenatal valproic acid (VPA) exposure and mutations in *fmr1*, *mecp2*, and *shank3* exhibit disrupted inhibitory transmission and behavioral traits akin to those of ASD (Souza et al., 2024). Preclinical studies have demonstrated the significance of ρ subunits in the pathophysiology of ASD. Specifically, revealed the dysregulation of the GABA_A_ρ3 (GABRR3) subunit within the VPA-induced autism model, thereby suggesting its involvement in altered inhibitory neurotransmission and neurodevelopmental consequences (Varman et al., 2018; Saavedra-Bonilla et al., 2024). Collectively, these observations indicate that GABA receptors containing ρ subunits may contribute to the E/I imbalance that is a hallmark of ASD, thereby representing a novel avenue for both mechanistic investigation and therapeutic intervention.

In ASD, impairments in inhibitory signaling extend beyond receptor-level alterations and involve GABA synthesis, transport, and degradation. Reduced expression of GAD1/GAD2, mutations in SLC6A1 (which encodes the GAT1 transporter), and developmental abnormalities in interneurons all contribute to impairing inhibitory signaling (Fatemi et al., 2002; Fatemi et al., 2009; Marilovtseva et al., 2025; Al Dera, 2022). Moreover, immune activation, oxidative stress, and neuroinflammation further affect GABAergic pathways, suggesting that molecular disruptions contribute to ASD-related inhibitory deficits (Al Dera, 2022). Although these insights enhance our understanding, creating effective treatments remains challenging. Broad GABAergic drugs such as benzodiazepines and barbiturates can cause sedation, tolerance, and developmental issues. This highlights the importance of developing targeted strategies that account for receptor subtypes, developmental stages, and neural circuits (Ghit et al., 2021). Future studies could investigate methods like neurosteroid modulation, chloride transporter regulation, and activity-driven circuit reorganization.

This review explores the role of GABA_A_ receptor dysfunction in the pathophysiology of ASD, with a focus on molecular alterations, inhibitory circuit abnormalities, and therapeutic implications. We discuss the structural and functional properties of GABA_A_ receptors, mechanisms of GABAergic dysfunction in ASD, subtype-specific receptor alterations, and evidence from pre-clinical and clinical studies. We also examined emerging pharmacological and non-pharmacological therapies targeting GABAergic signaling and explored current translational challenges along with prospects for precision neurotherapeutic strategies.

## 2 GABA_A_ receptors and inhibitory neurotransmission

GABA_A_ receptors are the primary mediators of rapid inhibitory neurotransmission in the mammalian CNS (Rosas-Arellano et al., 2023). Belonging to the Cys-loop ligand-gated ion channel superfamily, they regulate chloride (Cl^−^) ion flow across neuronal membranes, influencing synaptic integration, oscillatory activity, and the E/I balance (Olsen and Sieghart, 2009; Masiulis et al., 2019). These receptors are key to inhibitory circuit control and neurodevelopmental disorders, owing to their structural diversity and interaction with various drugs. GABA_A_ receptors are heteropentamers made up of 19 known human subunits: six α (α1–α6), three β (β1–β3), three γ (γ1–γ3), three ρ (ρ1–ρ3), plus single subunits δ, ε, π, and θ (Sigel and Ernst, 2018; Sieghart and Sperk, 2002; Laverty et al., 2019; Rosas-Arellano et al., 2023). Functional receptors consist of specific subunit combinations, usually including two α, two β, and one γ subunit. The most prevalent adult form in the brain is α1β2γ2, which assembles a core around the ion channel pore (Olsen and Sieghart, 2009; Laverty et al., 2019). Each subunit has a large external N-terminal domain that binds ligands, four transmembrane segments (TM1–TM4), and an intracellular loop located between TM3 and TM4. This loop plays a role in controlling receptor trafficking, phosphorylation, and interactions with cytoskeletal proteins (Connolly et al., 1996; Moss and Smart, 2001). The subunit composition of GABA_A_ receptors critically determines their functional properties. Receptors containing α1 subunits are mainly associated with sedative and anticonvulsant effects, whereas α2-and α3-containing receptors are associated with anxiolytic effects. In contrast, α5-containing receptors are enriched in hippocampal regions and are involved in cognitive processing and synaptic plasticity. Extra synaptic δ-containing receptors mediate tonic inhibition and modulate neuronal network stability. Alterations in the distribution or function of these receptor subtypes may differentially contribute to ASD-related abnormalities in cognition, sensory processing and social behavior (Olsen and Sieghart, 2009; Farrant and Nusser, 2005; Brickley and Mody, 2012).

Since different receptor subtypes are found in specific brain regions and serve distinct functions, precisely regulating their subunit makeup could be an effective strategy to restore the E/I balance in neurodevelopmental disorders. Therefore, understanding GABA_A_ receptor structure and regulation is essential for developing targeted therapeutic strategies. Their molecular diversity and vulnerability to genetic and environmental influences make them vital mediators of inhibitory dysfunction related to ASD. Gaining insights into their subunit composition, developmental regulation, and receptor trafficking is critical to creating targeted GABAergic treatments.

## 3 GABAergic impairment in ASD

Growing evidence from molecular, neurochemical, genetic, and neuroimaging studies indicates that GABAergic system disruption plays a significant role in ASD (Rubenstein and Merzenich, 2003; Biria et al., 2023; Gordon et al., 2026). Multiple lines of evidence from genetics, *postmortem* examinations, neuroimaging, and experimental studies highlight GABAergic disruption as a critical contributor to the underlying pathophysiology of ASD. GABA is essential for regulating neuronal excitability, synaptic function, cortical rhythms, and plasticity driven by experience (Ben-Ari, 2002; Cellot and Cherubini, 2014). Alterations in GABAergic signaling are central to the E/I imbalance implicated in ASD pathophysiology (Nelson and Valakh, 2015; Rubenstein and Merzenich, 2003). GABAergic signaling involves multiple processes, including synthesis, vesicular release, receptor binding, chloride homeostasis, and synaptic reuptake (Marilovtseva et al., 2025; Ghit et al., 2021). Disruptions at any stage of this pathway could diminish inhibitory tone and hinder the maturation of neuronal circuits. Importantly, GABAergic dysfunction correlates with abnormalities in glutamatergic transmission, intracellular signaling cascades (PI3K/AKT/mTOR, MAPK/ERK, and CREB/BDNF signaling cascades), and immune-related pathways associated with ASD (Jiang et al., 2022; Al Dera, 2022).

A consistent molecular finding in ASD is decreased activity of GAD, the enzyme that converts glutamate into GABA (Fatemi et al., 2002; Yip et al., 2007; Jiang et al., 2022). GAD1 and GAD2 encode the GAD67 and GAD65 isoforms, respectively, which contribute to GABA biosynthesis. GAD67 mainly maintains resting GABA levels in the cytoplasm, while GAD65 is located at presynaptic terminals and facilitates GABA production during neuronal activity (Soghomonian and Martin, 1998; Marilovtseva et al., 2025). From a developmental perspective, decreased GAD1 expression in human brain regions may have other implications beyond the reduction in neurotransmitter availability (Fatemi et al., 2002; Yip et al., 2007; Oblak et al., 2011). Because GABA acts as an excitatory trophic signal in early development due to high intracellular chloride, reduced GABA production during embryogenesis may disrupt interneuron migration, cortical layering, and synaptic maturation (Ben-Ari, 2002; Kaila et al., 2014; Braat and Kooy, 2015; Marilovtseva et al., 2025).

Neurochemical studies suggest widespread GABAergic imbalance in ASD, though findings differ across regions and developmental stages (Thompson, 2024; DeMayo et al., 2021). Early biochemical research identified reduced GABA concentrations in the plasma and CSF of individuals with ASD (Dhossche et al., 2002). Proton magnetic resonance spectroscopy (^1^H-MRS) has been validated *in vivo* for region-specific GABA alterations. Gaetz et al. (2014) documented reduced GABA levels in the sensorimotor cortex of children with ASD, whereas altered GABA concentrations were observed in the auditory and frontal brain regions (Puts et al., 2017), with the effects depending on age and sensory phenotype. Thompson (2024) reported distinct brain regions (hippocampus, cortex and thalamus) and age-dependent variability in GABA_A_ receptor-mediated inhibition, reflecting receptor subunit compositions (α, β, γ, and δ isoforms) and functional properties. A decline in cortical GABA levels is closely linked to disturbances in gamma-band oscillations, which rely on the synchronized firing of fast-spiking parvalbumin-positive (PV+) interneurons (Sohal and Rubenstein, 2019). Such disruptions are common in ASD and are thought to result from impaired inhibitory synchronization. Furthermore, substantial evidence suggests that genetic differences in GABA_A_ receptor subunit genes, combined with alterations in GABA production, elevate the likelihood of developing ASD (Cook et al., 1998; Ma et al., 2005; Jiang et al., 2022).

Epigenetic mechanisms also play a role in receptor dysfunction. Chao et al. (2010) showed that loss of *mecp2* disrupts GABAergic transmission and receptor expression *in vivo*. Modifications in the composition of receptor subunits may yield varying impacts on phasic and tonic inhibition. Synaptic α1–3/γ2-containing receptors control fast inhibitory transmission, while extrasynaptic α4–6/δ-containing receptors govern tonic inhibitory tone (Braat and Kooy, 2015; Ghit et al., 2021). Pharmacological studies on α5-preferring modulators support the importance of receptor subtype-specific strategies (Cecere et al., 2025).

GABAergic interneurons, especially PV+ fast-spiking basket and chandelier cells, play a vital role in generating and synchronizing cortical rhythms (Buzsáki and Wang, 2012). Various studies have shown decreased PV expression and interneuron abnormalities in ASD models (Gogolla et al., 2009; Filice et al., 2020). When PV+ interneurons fail to mature correctly or exhibit reduced activity, it results in insufficient inhibition, increased local connectivity, and disrupted long-range communication (Sohal and Rubenstein, 2019).

Rubenstein and Merzenich (2003) suggested that even a small decrease in inhibition during key stages of development can lead to persistent alterations in neural circuit organization and function. The evidence pointing to ASD includes reduced GABA synthesis, changes in extracellular GABA, altered receptor subunit composition and interneuron dysfunction, and chloride imbalance. This indicates that ASD involves a complex disruption in inhibitory neurotransmission rather than a simple, widespread deficit (Thompson, 2024; Jiang et al., 2022). GABAergic dysfunction interacts with other ASD-related pathways such as mTOR signaling, synaptic pruning, immune regulation, and calcium signaling by altering E/I balance, which has been shown to dysregulate mTOR-dependent synaptic protein synthesis, impair microglia-mediated synaptic pruning, enhance neuroinflammatory cytokine release, and disrupt calcium-dependent neurotransmission (Tang et al., 2014; Xiong et al., 2023). Therefore, GABAergic abnormalities should be seen as part of an integrated network of biological systems. Future therapies might focus on targeting specific receptor subtypes, boosting interneuron activity, restoring chloride balance, or adjusting GABA synthesis according to developmental stages (Souza et al., 2024).

## 4 Molecular subtypes of GABA_A_ receptors: alterations in ASD

The individuals with ASD show decreased levels of α1, α2, β3, and γ2 subunits in the prefrontal cortex and associated networks, though findings vary depending on the cohort and specific region examined (Mendez et al., 2013; Ajram et al., 2019). These brain regions are critical for executive function, emotional regulation, motor control, and social cognition, which are commonly impacted in ASD. The β3 subunit, encoded by the *GABRB3* gene on chromosome 15q11–q13, is one of the most consistently linked regions to ASD (Grove et al., 2019; Satterstrom et al., 2020). Large-scale exome sequencing and genome-wide association studies have confirmed that GABAergic pathway genes, including *GABRB3*, play a role in ASD risk, though their impact varies among different populations (Grove et al., 2019; Satterstrom et al., 2020). Since β3-containing receptors are crucial for inhibitory synaptic transmission, altered expression could weaken inhibitory currents and destabilize cortical microcircuits. Variations in γ2 and δ subunits suggest subtype-specific vulnerabilities: γ2 receptors are predominantly synaptic and mediate rapid phasic inhibition, whereas δ-containing receptors are extrasynaptic and mediate tonic inhibition (Sigel and Ernst, 2018).

Disruptions in γ2 expression can impair receptor clustering and synaptic stability, while alterations in δ-containing receptors might decrease tonic inhibitory control (Ajram et al., 2019). These alterations could play a role in the E/I imbalance seen in ASD (Nelson and Valakh, 2015). Changes in synaptic subunits can affect the timing of inhibitory signals, and disruptions in extrasynaptic subunits may compromise neuronal gain control and neural network stability. These subtype-specific disruptions are consistent with findings of altered cortical connectivity and microcircuit organization seen in ASD (Uddin et al., 2013; Nelson and Valakh, 2015). Reduced synaptic inhibition could lead to increased local hyperconnectivity, while decreased tonic inhibition may lower seizure thresholds and raise cortical excitability.

**FIGURE 1:**
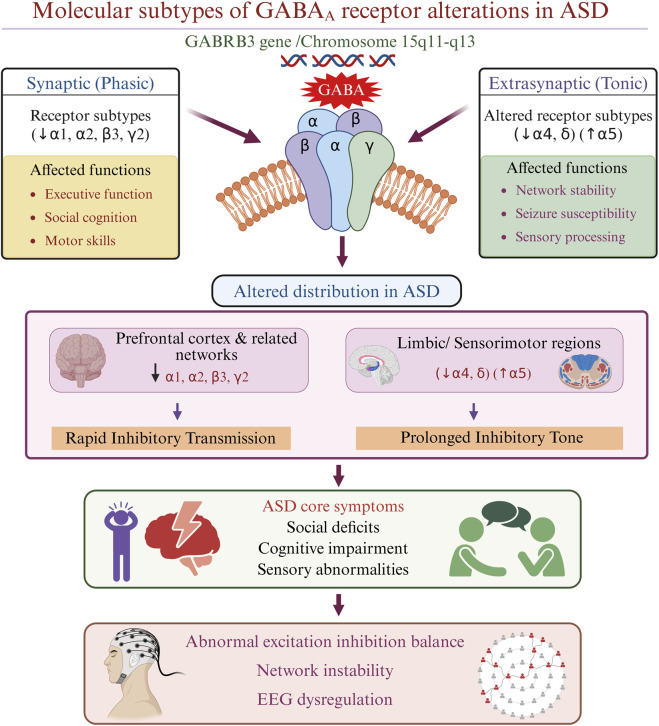
Molecular subtypes of GABA_A_ receptor alterations in autism spectrum disorder (ASD). The diagram illustrates how synaptic (phasic) and extrasynaptic (tonic) GABA_A_ receptors are impacted in ASD. Alterations in subunit composition, particularly reductions in α1, α2, β3, and γ2 subunits, disrupt rapid inhibitory signaling in cortical networks. Additionally, changes in α4, α5, and δ subunits of extrasynaptic receptors affect tonic inhibition, mainly in limbic and sensorimotor regions. Variations in receptor distribution between the prefrontal cortex and limbic circuits disturb the E/I balance, leading to network instability, EEG irregularities, hyperactivity or hypoactivity, and core ASD features such as social impairments and cognitive difficulties.

Disruption in chloride homeostasis or delays in its developmental switch may hinder cortical circuit development, resulting in a lasting imbalance between excitatory and inhibitory signals, a key theory in ASD (Tyzio et al., 2014; Ben-Ari, 2002). In early development, NKCC1 keeps intracellular chloride high, making GABA depolarizing; later, increased KCC2 levels turn GABA into an inhibitory hyperpolarizing signal (Kaila et al., 2014; Tyzio et al., 2014). If this chloride switch during development is disrupted, it has been linked to neurodevelopmental disorders such as ASD (He et al., 2014). Depolarizing GABA signaling may disrupt neuronal migration, dendrite growth, and synapse formation, ultimately affecting neural circuit development. Alterations in inhibitory interneuron populations, particularly PV^+^ interneurons, have been reported in ASD (as discussed in Section 3). These findings align with observed impairments in working memory, executive function, and social processing in individuals with ASD. Pharmacological targeting of specific GABA_A_ receptor subtypes represents a promising therapeutic strategy. These results favor a precision pharmacology approach over general inhibition. Taken together, converging genetic, molecular, imaging, and circuit-level evidence suggests that ASD involves subtype-specific disruptions of GABA_A_ receptors rather than uniform global deficiency (Figure 1).

## 5 Preclinical evidence: animal models of GABA_A_ malfunction in ASD

Preclinical studies demonstrate that changes in GABA_A_ receptor subunits are linked to reduced inhibitory postsynaptic currents, decreased receptor clustering, and destabilization of synaptic scaffolding proteins such as gephyrin; these changes subsequently lead to impaired inhibitory signaling and circuit hyperexcitability (Han et al., 2012; Tyzio et al., 2014; Chu et al., 2022). Furthermore, autism-like models exhibit a reduction in GABAergic neuron populations, decreased expression of GAD65/67, and altered inhibitory synaptic transmission within hippocampal and cortical circuits, which are associated with deficits in social behavior and cognitive function (Peñagarikano et al., 2011; Jurkovičová-Tarabová et al., 2025). Collectively, these results imply that a reduction in inhibitory activity alone can trigger ASD-like network dysfunction. This includes hyperexcitability, altered synaptic plasticity, and impaired long-range connectivity. This aligns with the E/I imbalance observed in both genetic and environmental models (Han et al., 2012; Peñagarikano et al., 2011; Sacai et al., 2020).

### 5.1 Environmental models of GABAergic dysfunction

VPA is an antiepileptic drug and a histone deacetylase inhibitor, one of the most studied ASD models (Chomiak et al., 2013). Prenatal exposure to VPA during critical developmental windows is induces ASD-like behavioral phenotypes, including reduced social interaction, repetitive behaviors, anxiety-like responses, and impairments in learning and memory (Chomiak et al., 2013; Nicolini and Fahnestock, 2018). VPA significantly influences GABAergic pathways in the brain (Chomiak et al., 2013; Nicolini and Fahnestock, 2018). Mechanistically, VPA exposure alters histone acetylation and transcriptional regulation of GABAergic genes, resulting in reduced expression of GABA_A_ receptor subunits, impaired receptor clustering, and disrupted inhibitory synaptic maturation.

VPA-induced ASD models show reduced GAD65/67 expression and impaired PV^+^ interneuron function (see Section 3). These findings collectively indicate reduced inhibitory synaptic transmission and cortical hyperexcitability (Al Sagheer et al., 2018; Alruwaili et al., 2026).

Electrophysiological investigations reveal that male juvenile rats exposed to VPA demonstrate diminished inhibitory postsynaptic currents within the prefrontal cortex and hippocampus. Furthermore, these rats exhibit alterations in gamma-band oscillations, specifically within the 30–80 Hz range. These findings provide support for the association between compromised GABA_A_-mediated inhibition and the manifestation of behaviors analogous to those observed in ASD (Nicolini and Fahnestock, 2018; Wang et al., 2018). Benzodiazepines, like diazepam and clonazepam, and direct agonists such as muscimol, which enhance GABA_A_ receptor activity, increase inhibitory neurotransmission. This effect has been shown to improve social interaction and cognitive function in animal models of ASD (Han et al., 2012; Souza et al., 2024).

Maternal immune activation (MIA), caused by administering polyinosinicpolycytidylic acid to pregnant females, leads to persistent alterations in interneuron development and GABA-related gene expression (Canetta et al., 2016; Baines et al., 2020). Studies show reduced GAD67 expression, impaired interneuron migration, and abnormal cortical layering, which collectively result in disrupted inhibitory circuit formation and long-lasting E/I imbalance in the offspring cortex (Baines et al., 2020; Canetta et al., 2016). Offspring exposed to MIA display reduced inhibitory activity and disrupted oscillatory synchronization, especially in frontal brain regions responsible for executive and social functions (Estes and McAllister, 2016). These neurophysiological alterations are associated with challenges in social recognition, increased anxiety, and repetitive behaviors (Schneider Gasser et al., 2024). Taken together, prenatal inflammatory disruptions may cause GABA_A_ receptor dysfunction and disturb the E/I balance (Baines et al., 2020; Estes and McAllister, 2016).

### 5.2 Genetic models illustrating the role of GABA_A_ receptors

Studies using genetically modified mouse models that target high-confidence ASD risk genes consistently show widespread GABAergic dysfunctions (Zhao et al., 2022; Wang and Sun, 2025; Giniatullin and Cherubini, 2025). Despite the distinct biochemical functions of these genes, they all impact inhibitory circuit health and synapse functionality (Satterstrom et al., 2020). Models lacking *shank3* modifications, with *shank3* being a scaffolding protein crucial for organizing the postsynaptic density, are strongly linked to ASD and Phelan-McDermid syndrome (Monteiro and Feng, 2017). Mice deficient in *shank3* show decreased levels of specific GABA_A_ receptor subunits, especially α2, which impairs their cortico-striatal circuit’s ability to transmit inhibitory signals (Bey et al., 2018; Vicidomini et al., 2017). *Shank3* deficiency disrupts postsynaptic scaffolding proteins that stabilize GABA_A_ receptor localization and inhibitory synapse organization, thereby impairing inhibitory neurotransmission. These mice may exhibit social challenges, excessive grooming, and unease (Bey et al., 2018). *Mecp2*-mutations associated with Rett syndrome produce substantial impairments in inhibitory neurotransmission and interneuron function (Chao et al., 2010). *Mecp2* mutations also alter transcriptional regulation of genes involved in GABA synthesis and receptor expression, contributing to reduced inhibitory signaling. Genetic ASD models, including *shank3, mecp2,* and *fmr1* mutations, consistently exhibit GABAergic deficits, including impaired inhibitory transmission and interneuron dysfunction (see Section 3). These animals exhibit increased seizure susceptibility and heightened network excitability (Curia et al., 2009). Enhancing GABAergic transmission in these animals markedly reduces hyperactivity and improves behavior, emphasizing the importance of inhibitory circuit deficits in ASD-related conditions (Han et al., 2012).

Together, genetic and environmental models consistently demonstrate impaired GABAergic inhibition and disrupted cortical network synchronization. Both environmental and genetic ASD models exhibit similar neurophysiological traits, including reduced synaptic inhibition, increased cortical excitability, and disrupted network oscillations (Han et al., 2012; Goffin et al., 2011). These alterations reflect disrupted inhibitory circuit function (see Section 3). The decrease in inhibitory drive makes it harder for GABA_A_ receptors to relay information between neurons, reduces inhibitory postsynaptic currents, and disrupts oscillatory synchronization (Wang et al., 2013; Gogolla et al., 2009). This deficiency disrupts neuronal synchronization and destabilizes key brain networks crucial for cognition and social behavior (Gogolla et al., 2009). Ongoing inhibitory deficits, which can often be reversed with specific medications, support the notion that GABAergic dysfunction is a core, rather than secondary, contributor to ASD traits (Han et al., 2012). Preclinical studies indicate that disrupted GABA_A_ receptor signaling leads to circuit imbalances and behavioral problems in ASD models (Satterstrom et al., 2020). Approaches that boost inhibitory signaling offer a logical, mechanism-driven strategy for alleviating ASD symptoms. To improve clarity and summarize key mechanistic findings, the major preclinical studies demonstrating GABA_A_ receptor dysfunction in ASD models are summarized in Table 1.

**TABLE 1:** Summary of preclinical evidence demonstrating GABA_A_ receptor dysfunction in ASD models.

ASD model	GABAergic alteration	Brain region	Behavioral phenotype	Key findings/implications	References
VPA model	Reduced GAD65/67 expression, impaired PV+ interneurons, reduced IPSCs	Prefrontal cortex, hippocampus	Social deficits, repetitive behavior, anxiety	Reduced inhibitory transmission and cortical hyperexcitability	Al Sagheer et al., 2018; Wang et al., 2018
Maternal immune activation	Reduced GAD67, impaired interneuron migration	Frontal cortex	Anxiety, repetitive behavior, social impairment	Prenatal inflammation disrupts inhibitory circuit development.	Canetta et al. (2016)
Shank3 knockout	Reduced α2 GABA_A_ receptor subunits	Cortico-striatal circuits	Social deficits, excessive grooming	Impaired inhibitory signaling and synaptic dysfunction	Bey et al. (2018)
Mecp2-null model	Reduced PV+ interneuron activity, altered receptor expression	Cortex	Rett-like autistic behaviors	Reduced inhibitory synaptic transmission	Chao et al. (2010)
Fmr1 knockout	Reduced GABA_A_ receptor subunits, weak inhibitory signaling	Hippocampus, cortex	Hyperexcitability, seizure susceptibility	Deficient inhibitory network regulation	Curia et al. (2009)

ASD, autism spectrum disorder; VPA, valproic acid; GABA, Gamma-aminobutyric acid; GABA_A_, Gamma-aminobutyric acid type A; GAD65/67, Glutamic acid decarboxylase 65/67; PV+, Parvalbumin-positive; IPSCs, Inhibitory postsynaptic currents; Shank3, SH3 and multiple ankyrin repeat domains 3; Mecp2, Methyl-CpG-binding protein 2; Fmr1, Fragile X messenger ribonucleoprotein 1.

## 6 Clinical evidence: GABAergic indicators and neuroimaging in ASD

*Postmortem* studies show decreased levels of GAD65/67 in cortical and cerebellar tissues through the Western blot technique (Fatemi et al., 2002; Yip et al., 2007), along with altered GABA_A_ receptor subunit binding in the autistic brain (Oblak et al., 2011). In addition, *in vivo* magnetic resonance spectroscopy shows that individuals with ASD have reduced cortical GABA levels (Gaetz et al., 2014).

The E/I imbalance hypothesis proposes that impaired inhibitory signaling disrupts cortical synchronization, sensory processing, and higher cognitive functions in ASD (Colomar et al., 2023; Nelson and Valakh, 2015). Advances in neuroimaging techniques have enabled *in vivo* investigation of GABAergic alterations in individuals with ASD. ^1^H-MRS and positron emission tomography (PET) provide complementary approaches for assessing GABA concentrations and GABA_A_ receptor availability, respectively (Du et al., 2023; Horder et al., 2018).

^1^H-MRS studies consistently report reduced cortical GABA levels in individuals with ASD, particularly in the anterior cingulate cortex, prefrontal cortex, sensorimotor cortex, and auditory cortex, regions involved in executive function, social cognition, and sensory processing (Du et al., 2023; Song et al., 2024). These neurochemical alterations appear to vary across developmental stages, suggesting that disrupted maturation of inhibitory circuits may affect critical periods of cortical plasticity (DeMayo et al., 2021; Song et al., 2024). Moreover, lower GABA concentrations measured by ^1^H-MRS have been associated with behavioral characteristics such as sensory hypersensitivity, repetitive behaviors, and impaired social communication (Song et al., 2024). Collectively, these findings indicate that reduced inhibitory signaling may contribute to abnormal cortical excitability and disrupted network coordination in ASD. To further understand these alterations, neuroimaging and *postmortem* studies provide complementary evidence regarding GABAergic dysfunction in ASD.

### 6.1 Neuroimaging evidence

PET imaging studies have demonstrated altered availability of GABA_A_ receptors in individuals with ASD (Horder et al., 2018; Mendez et al., 2013). PET imaging using α5-selective radioligands shows alterations in GABA_A_ receptor availability in limbic regions of adults with ASD (Horder et al., 2018). Radioligands such as [^11^C] flumazenil, which selectively bind to the benzodiazepine site of GABA_A_ receptors, have revealed decreased binding potential in the frontal and temporal cortices of individuals with ASD (Mendez et al., 2013). Reduced ligand binding may reflect decreased receptor density, altered receptor subunit composition, or changes in receptor affinity (Horder et al., 2018; Mendez et al., 2013). Because the composition of receptor subunits affects both pharmacological properties and functional signaling, even subtle changes in subunit distribution may substantially affect inhibitory neurotransmission and cortical oscillatory activity (Laverty et al., 2019).

### 6.2 *Postmortem* molecular findings

*Postmortem* examinations corroborate these neuroimaging findings, demonstrating structural and molecular irregularities within the GABAergic system of individuals with ASD (Hashemi et al., 2017; Amina et al., 2021). Numerous studies have documented diminished expression of various GABA_A_ receptor subunits, encompassing α1, α2, α3, β2, β3, and γ2, within the hippocampus and prefrontal cortex. These areas are essential for memory, executive function, and social conduct (Fatemi et al., 2014; Amina et al., 2021; Fateimi et al., 2024). In addition, decreased expression of GAD isoforms (GAD65 and GAD67) has been observed in both cortical and subcortical regions (Chattopadhyaya et al., 2007; Yip et al., 2007), indicating impaired GABA synthesis. Alterations in interneuron populations, particularly PV^+^ interneurons, have also been documented in ASD brain tissue (Hashemi et al., 2017; Amina et al., 2021).

### 6.3 Clinical implications and biomarker potential

The association between GABAergic abnormalities and symptom severity in ASD underscores their clinical relevance. Lower cortical GABA levels and reduced receptor binding have been linked to impairments in social communication, language development, and cognitive flexibility (Song et al., 2024). Furthermore, inhibitory dysfunction may contribute to common ASD comorbidities such as epilepsy, anxiety, and sleep disturbances, which are often associated with disrupted E/I balance (Nelson and Valakh, 2015).

Neuroimaging, molecular, and neuropathological studies suggest that ASD involves widespread alterations in inhibitory signaling networks (Du et al., 2023; Parrella et al., 2024; Yan et al., 2026). These disruptions affect multiple cortical and limbic regions and involve abnormalities in neurotransmitter synthesis, receptor expression, and interneuron function. Such findings support the view of ASD as a network disorder involving dysregulated synaptic signaling pathways (Gandal et al., 2018). Table 2 summarizes the major clinical and neuroimaging findings that support GABAergic dysfunction in ASD.

**TABLE 2:** Clinical evidence supporting GABAergic dysfunction in individuals with ASD.

Clinical approach	Findings	Brain region	Clinical relevance	References
^1^H-MRS	Reduced cortical GABA levels	ACC, frontal cortex, sensorimotor cortex	Associated with sensory and social deficits	Gaetz et al., 2014; Song et al., 2024
PET imaging ([^11^C] flumazenil)	Reduced GABA_A_ receptor binding	Frontal and temporal cortex	Suggests altered receptor availability	Mendez et al. (2013)
*Postmortem* studies	Reduced α1, β2, β3, and γ2 subunits	Hippocampus, prefrontal cortex	Impaired inhibitory signaling	Fatemi et al., 2013
GAD65/67 expression studies	Reduced GABA synthesis enzymes	Cortical and cerebellar tissue	Reduced inhibitory neurotransmission	Yip et al. (2007)
PV+ interneuron analyses	Reduced PV+ interneuron density	Prefrontal cortex	Altered gamma oscillations and E/I balance	Hashemi et al. (2017)

ASD, autism spectrum disorder; GABA, Gamma-aminobutyric acid; GABA_A_, Gamma-aminobutyric acid type A; ^1^H-MRS, proton magnetic resonance spectroscopy; PET, positron emission tomography; ACC, anterior cingulate cortex; PV+, Parvalbumin-positive; E/I, Excitation/inhibition.

Importantly, biomarkers derived from GABA-related measures, such as regional GABA concentrations detected by MRS or receptor binding assessed by PET, may help stratify patient subgroups and monitor treatment responses (Du et al., 2023; Horder et al., 2018). Future research integrating multimodal neuroimaging, genetic profiling, and developmental analyses will be essential for determining whether GABA-related biomarkers can predict clinical outcomes and guide therapeutic strategies (Satterstrom et al., 2020). These alterations contribute to cortical hyperexcitability and network instability, emphasizing the possible therapeutic value of interventions aimed at restoring inhibitory homeostasis in ASD (Souza et al., 2024).

## 7 Pharmacological modulation of GABA_A_ receptors

GABAergic abnormalities are clinically important because they are associated with the severity of symptoms. Reduced cortical GABA levels and diminished receptor binding are associated with increased difficulties in social reciprocity, language, and cognitive flexibility (Rojas et al., 2014; Oblak et al., 2009; Oblak et al., 2011; Fung et al., 2021).

Traditional benzodiazepines act as positive allosteric modulators at the α–γ interface of GABA_A_ receptors, enhancing inhibitory currents. However, their non-selective activation, particularly of α1 subunits, often causes sedation, tolerance, and cognitive impairment, limiting long-term use in ASD (Ghit et al., 2021; Jiang et al., 2022; Al Dera, 2022).

To overcome these limitations, researchers are developing benzodiazepine-site ligands that selectively target α2-and α3-containing GABA_A_ receptors, mainly linked to reducing anxiety and preventing seizures while minimizing α1-related sedation (Ghit et al., 2021; Goldschen-Ohm, 2022). Preclinical studies also indicate that focusing on receptor subtypes may provide therapeutic advantages. For example, positive allosteric modulation of α5-containing GABA_A_ receptors has enhanced social and cognitive functions in rodent autism models triggered by valproic acid, without worsening dopaminergic hyperactivity (Souza et al., 2024). Similarly, the α5-selective positive allosteric modulator SH-053–2’F-R-CH3 improved social interaction and recognition memory in a valproic acid-induced rodent model of ASD, without inducing locomotor hyperactivity or sedation (Schiavi et al., 2019). In addition, alogabat, a selective GABA_A_-α5 positive allosteric modulator, ameliorated social behavior, cognitive flexibility, and network synchronization in preclinical neurodevelopmental models with impaired inhibitory signaling (Cecere et al., 2025). Neurosteroid-based modulators such as ganaxolone, a synthetic analogue of allopregnanolone, showed efficacy in restoring tonic inhibition and reducing hyperexcitability in disorders associated with GABAergic dysfunction (De, 2024). Furthermore, bumetanide-induced modulation of NKCC1/KCC2-mediated chloride homeostasis has been shown to improve social responsiveness and reduce repetitive behaviors in some clinical studies in ASD, although variability in treatment response remains a challenge (Lemonnier et al., 2012; Fuentes et al., 2023). These findings indicate that modulating GABA_A_ receptor signaling in a circuit- and subtype-specific manner may have therapeutic potential for ASD.

Subtype-selective modulation of GABA_A_ receptors has furnished crucial experimental support for distinguishing anxiolysis from sedation. Atack (2008) showed that TPA023 binds with high affinity to GABA_A_ receptors containing α1, α2, α3, and α5 subunits; however, it demonstrates partial agonist efficacy primarily at α2/α3 subtypes, while exhibiting minimal efficacy at α1-containing receptors. Functionally, TPA023 elicited significant anxiolytic-like effects in rodent models, encompassing the elevated plus maze, fear-potentiated startle, and conditioned suppression tests, at doses that did not cause sedation or motor impairment in rotarod and operant behavioral assessments (Atack et al., 2011). L-838,417 acts as an agonist at α2-, α3-, and α5-containing receptors. However, it acts as an antagonist at α1-containing receptors (Rowlett et al., 2005). In studies with non-human primates, L-838,417 showed effects similar to those of anxiolytics in conflict situations. However, it caused fewer sedative and ataxic effects compared to standard benzodiazepines. These findings indicate that the α2/α3 subunits are important for the mechanisms underlying anxiolysis. Furthermore, studies using electrophysiology have shown that allopregnanolone, a naturally occurring neurosteroid, enhances the function of GABA_A_ receptors, even when the binding of GABA is limited (Bitran et al., 1995). These findings indicate a different way of working than how benzodiazepines usually work. Neurosteroids bind within inter-subunit sites of the transmembrane domain, particularly at the β-subunit negative interface (Middendorp et al., 2014). This supports the idea that these compounds enhance receptor function without using the usual benzodiazepine binding site. However, both pharmacological and genetic evidence consistently show that α1-containing GABA_A_ receptors are involved in sedation and tolerance. This limits the long-term use of non-selective GABAergic agents in children and those with neurodevelopmental disorders (McKernan et al., 2000; Rudolph and Möhler, 2006). Side effects like sedation, cognitive impairment, behavioral disinhibition, and dependence are notable (Ghit et al., 2021). Furthermore, non-specific potentiation could disrupt normal brain rhythms essential for cognition (Jiang et al., 2022). To address this issue, researchers are working on benzodiazepine-site ligands that selectively target α2-and α3-containing GABA_A_ receptors, which are mainly associated with anxiety reduction and seizure control, while avoiding α1-mediated sedation (Ghit et al., 2021; Engin, 2023).

In rodent models of ASD induced by valproic acid, α5-selective positive allosteric modulators alleviated social and recognition memory issues without worsening dopaminergic imbalances, suggesting that hippocampal α5 receptor modulation can enhance cognition while preserving overall neurotransmitter balance (Mehra et al., 2022; Schiavi et al., 2019; Vasconcelos et al., 2025). These developments point to a shift from broad inhibitory enhancement to precise circuit repair targeting specific receptor subtypes (Antoine et al., 2019; Nelson and Valakh, 2015). This aligns with current ASD models emphasizing synaptic and microcircuit problems over widespread neurotransmitter deficits (Jiang et al., 2022).

Although preclinical results are promising, several important translational challenges need resolution before GABA_A_ receptor–focused therapies can be widely used in ASD treatment (Tyzio et al., 2014; Lemonnier et al., 2012). Developmental timing is critical, as GABAergic signaling changes with age, including the well-known switch from excitatory to inhibitory chloride mediated by NKCC1 and KCC2 (Ben-Ari, 2002; Tyzio et al., 2014). Treatments during sensitive developmental periods should account for receptor distribution, chloride balance, and network development to avoid interfering with neurodevelopment (Tyzio et al., 2014). The variety of GABA_A_ receptor subunits adds complexity, necessitating subtype-specific approaches to prevent side effects from broad modulation (Rudolph and Möhler, 2006). Long-term safety, particularly for children, raises concerns since chronic GABA enhancement may lead to tolerance, receptor desensitization, hormonal shifts, or hindered synaptic development (Rudolph and Möhler, 2006; Ben-Ari, 2002). Furthermore, given the high heterogeneity of ASD, biomarker-based patient stratification is essential. Detecting molecular, genetic, or electrophysiological indicators of GABAergic dysfunction can help in choosing appropriate patients, fine-tuning dosages, and improving treatment outcomes (Fung et al., 2021; Jiang et al., 2022). These translational challenges highlight the importance of designing developmentally appropriate, precision approaches when applying GABA_A_ receptor modulators in ASD therapy (Tyzio et al., 2014; Fung et al., 2021).

In summary, pharmacological targeting of GABA_A_ receptors represents a promising and highly detailed approach to treating ASD (Rudolph and Möhler, 2006; Antoine et al., 2019). By targeting specific receptor subtypes and different modes of modulation, whether synaptic or extrasynaptic, new compounds seek to improve inhibitory balance more effectively while reducing side effects (Changeux and Christopoulos, 2017; Thompson, 2024). Ongoing research that combines molecular neuroscience, animal studies, and carefully planned clinical trials is crucial for turning these scientific developments into safe, effective treatments for ASD (Antoine et al., 2019; Tyzio et al., 2014).

## 8 Non-pharmacological approaches

Environmental enrichment (EE) refers to systematically providing heightened sensory, cognitive, motor, and social stimuli beyond normal conditions. In preclinical neuroscience, EE is frequently used to study how experiences affect brain adaptability in models of neurodevelopmental disorders like ASD (Li et al., 2024; Binder and Bordey, 2023; Flores-Prieto et al., 2024). Enrichment programs typically include increased physical activity, social engagement, exposure to new experiences, and problem-solving activities, all of which stimulate different neural circuits. At the cellular and molecular level, EE affects GABAergic function through several mechanisms. Studies indicate that enriched environments boost the expression of GAD isoforms, elevate GABA_A_ receptor subunit levels, and facilitate the maturation of inhibitory interneurons, especially PV^+^ interneurons (Ball et al., 2019; Burrows et al., 2015). These changes are vital for stabilizing cortical microcircuits and maintaining E/I balance.

In genetic models of ASD, such as *shank3* mutant mice, EE has been shown to restore inhibitory synaptic transmission and reduce behavioral abnormalities like social deficits and repetitive behaviors (Binder and Bordey, 2023; Li et al., 2024). Similarly, in rodent models induced by valproic acid, EE raises GABA receptor levels, enhances interneuron activity, and ameliorates anxiety-like behavior, social deficits, and cognitive inflexibility (Fereshetyan et al., 2021; Schiavi et al., 2019). These benefits likely originate from improved synaptic integration and more robust inhibitory connections in the hippocampus and prefrontal cortex (Vakilzadeh et al., 2024). Apart from molecular effects, EE induces structural modifications like enhanced dendritic branching, increased spine density in certain areas, and improved long-term potentiation (Li et al., 2024; Flores-Prieto et al., 2024). These structural changes are crucial for E/I balance, thus avoiding excessive synaptic activity. Collectively, EE provides a safe, system-wide method to alter neural circuits by activity-dependent regulation of GABAergic activity (Tatti et al., 2017; Cohen Kadosh, 2025; Wu et al., 2022). This highlights that neural circuit abnormalities in ASD are still adaptable and can be influenced by experience.

Sensory processing issues are a fundamental aspect of ASD, involving unusual sensory integration across cortical and subcortical regions (Suprunowicz et al., 2025; Song et al., 2024). Many early interventions aim to recalibrate sensorimotor circuits during critical developmental periods to support neural development. Common methods encompass occupational therapy, sensory integration therapy, structured play, and social communication training, all designed to improve behavior and social skills. From a neurobiological standpoint, these interventions likely work through activity-dependent plasticity mechanisms (Li et al., 2024; Madia et al., 2025). Repeated, structured sensory stimulation can improve synaptic refinement and help stabilize inhibitory circuits. Tactile, proprioceptive, and vestibular inputs play a crucial role in influencing cortical inhibitory networks by affecting thalamocortical connections and interneuron activity patterns (Jung et al., 2026; Vakilzadeh et al., 2024). Experimental evidence indicates that enriched sensorimotor experiences can increase GAD expression, strengthen inhibitory synaptic currents, and modify GABA_A_ receptor composition in the somatosensory and prefrontal cortices (Li et al., 2024; Madia et al., 2025).

Early intervention is important because the development of GABAergic interneurons and chloride balance is highly active during infancy and childhood (Warm et al., 2022; Le Magueresse and Monyer, 2013). Structured behavioral engagement during these periods can promote the proper development of inhibitory circuits, helping to prevent long-term E/I imbalance. Additionally, sensory-based therapies might reduce cortical hyperexcitability by boosting both tonic and phasic inhibition, which can improve emotional regulation and decrease repetitive behaviors (Song et al., 2024; Suprunowicz et al., 2025). Interventions such as Applied Behavior Analysis and the Early Start Denver Model can also indirectly promote inhibitory balance. Chronic stress and dysregulation of the hypothalamic-pituitary-adrenal axis (HPA) axis may disrupt GABAergic signaling, as experimental studies demonstrate that GABAergic inputs within the hypothalamic paraventricular nucleus exert inhibitory control over stress responses, while chronic stress impairs this inhibition and enhances HPA axis activation (Decavel and Van den Pol, 1990; Herman et al., 2016; Maguire, 2014). Using predictable, reinforcement-based therapies can create learning environments that reduce stress-related excitotoxicity and support positive changes in synapses, as experimental studies show that activity-dependent plasticity engages BDNF–TrkB signaling, CREB phosphorylation, and NMDA receptor–dependent synaptic strengthening (Karpova et al., 2011; Greer and Greenberg, 2008; Li et al., 2024; Binder and Bordey, 2023). While the specific molecular pathways are still being studied, existing evidence suggests that regular behavioral reinforcement and social activities foster neuroplasticity in the prefrontal and limbic areas through GABAergic interneuron regulation and E/I balance, supported by studies demonstrating experience-dependent remodeling of inhibitory circuits and synaptic connectivity (Donato et al., 2025; Sale et al., 2007; Boutitah-Benyaich et al., 2025; Fathallah et al., 2023).

Taken together, sensory and behavioral therapies highlight experience-dependent neuroplasticity through structured experiences. While these therapies do not directly target GABA_A_ receptors pharmacologically, they may enhance synaptic plasticity and improve behavioral outcomes in ASD (Madia et al., 2025; Li et al., 2024). Starting these interventions early and with sufficient intensity could offer neuroprotective effects by aiding circuit compensation and reducing long-term risks associated with E/I imbalance.

**FIGURE 2:**
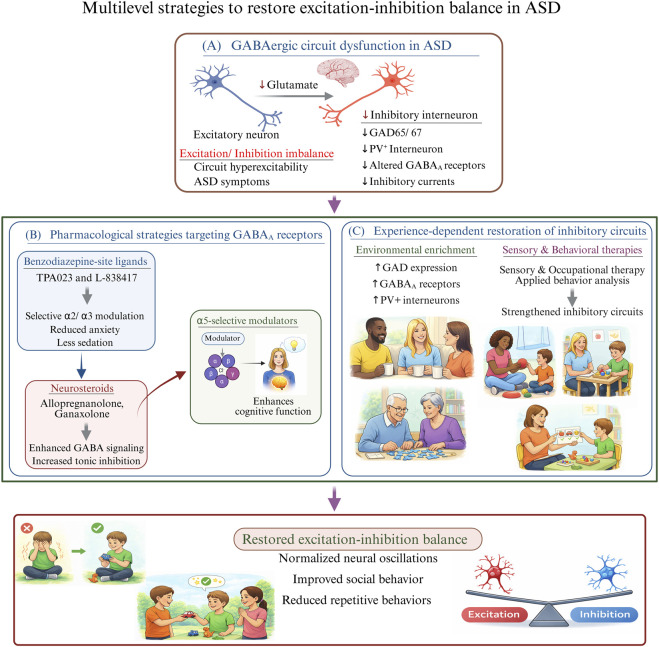
Multilevel strategies for restoring E/I balance in autism spectrum disorder. **(A)** In ASD, reduced GAD expression, impaired PV^+^ interneuron development, and altered GABA receptor subunit composition weaken inhibitory signaling, leading to E/I imbalance and circuit hyperexcitability in brain regions such as the prefrontal cortex, hippocampus, and amygdala. **(B)** Pharmacological strategies aim to restore inhibitory tone using subtype-selective benzodiazepine-site ligands (α2/α3), neurosteroid modulators (e.g., allopregnanolone, ganaxolone), and α5-selective compounds targeting cognitive circuits. **(C)** Non-pharmacological interventions, such as environmental enrichment and sensory-behavioral therapies, promote activity-dependent plasticity and strengthen inhibitory networks. Together, these approaches may help stabilize neural circuits and improve behavioral outcomes in ASD.

The restoration of E/I balance in ASD requires a multilevel therapeutic strategy that integrates molecular, pharmacological, and experience-dependent interventions targeting GABAergic circuits (Figure 2).

## 9 Therapeutic challenges and prospective directions

Despite progress in understanding the role of GABA_A_ receptors in ASD pathophysiology, creating effective treatments based on this research is still challenging (Giniatullin and Cherubuni, 2025). These challenges include the specific mechanisms by which GABA acts during development, the diverse structures and functions of GABA_A_ receptor subunits, the lack of reliable biomarkers to identify patients, and the limitations of current clinical trial methods. To develop effective neurotherapeutic strategies, it is crucial to overcome these challenges. A major challenge in using the GABAergic system to treat ASD is the age-related changes in how GABA works during brain development (Topchiy et al., 2024). Because GABA signaling changes as the brain develops, treatments need to consider the timing of the NKCC1–KCC2 switch. Therefore, using GABA_A_ receptor modulators during this important developmental stage requires careful thought. Intervening prematurely, whether through the augmentation of inhibitory processes or the suppression of naturally occurring depolarizing GABA effects, could disrupt the activity-dependent circuits that govern cortical development. On the other hand, delaying the correction of inhibitory deficits could lead to harmful changes in neural networks, which would then make it harder to reverse these changes later.

To determine the best timing for treatments, it is crucial to analyze brain activity and molecular changes over time, using both animal models and human subjects. The different types of GABA_A_ receptors are created by the combination of five different subunits. These subtypes are characterized by their anatomical distribution, biological roles, and responses to drugs (Ghit et al., 2021). Alterations in particular subunits have been associated with decreased tonic and phasic inhibition in ASD and other neurodevelopmental disorders (Absalom et al., 2024). Many existing drugs that work on the GABA system, including traditional benzodiazepines, do not specifically target certain receptor subtypes. Instead, these substances usually affect synaptic γ2-containing receptors, which can lead to several side effects. Therefore, creating modulators that specifically target certain receptor subtypes is essential to reduce these negative effects. Much attention has been directed toward extrasynaptic δ-subunit-containing receptors that modulate tonic inhibition and contribute to network stability (Arslan, 2021). Similarly, α5-containing receptors, mostly located in hippocampal and cortical areas, are promising targets for treating cognitive and social deficits (Souza et al., 2024; Cecere et al., 2025). Developing highly selective positive or negative allosteric modulators could enable fine-tuned regulation of the E/I balance without impairing overall neural activity. ASD presents with diverse clinical and biological features, making it challenging to find reliable biomarkers for GABAergic dysfunction or to forecast treatment outcomes. Current diagnostics mainly depend on behavioral assessments, which overlook underlying neurochemical or circuit alterations. Advanced neuroimaging techniques can measure GABA levels in specific brain regions of living individuals. Although useful, these techniques face challenges like limited spatial resolution, variability in protocols, and inconsistent results (Thomson et al., 2024; Saleh et al., 2024).

Additionally, measuring regional GABA concentrations by itself does not accurately show the distribution of receptor subtypes or distinguish between synaptic and extrasynaptic activity. Future biomarker strategies should integrate multimodal techniques, including electrophysiological signatures like gamma oscillations to assess cortical inhibition, transcriptomic profiles, peripheral molecular markers, and genetic risk factors related to GABA pathway components (Arutiunian et al., 2024; Hollestein et al., 2023). Combining these methods can improve biologically based patient stratification and simplify the interpretation of clinical trial results. Although several GABA-modulating agents have shown promising results in animal models of ASD, clinical outcomes have been variable. Bumetanide inhibits NKCC1 to restore GABA’s inhibitory function by regulating chloride levels, with promising early outcomes. Nonetheless, differences in effect sizes, safety issues with long-term use, and conflicting findings emphasize the necessity of comprehensive, well-designed randomized controlled trials (Fuentes et al., 2023; Hendi et al., 2026). Many current studies have limitations like small sample sizes, inconsistent inclusion criteria, short intervention periods, and reliance on subjective measures. Collectively, excluding participants based on biological markers might overlook responses unique to specific subgroups. Future research should adopt stratified trial designs. These study designs should include genetic, neurophysiological, or molecular markers. Furthermore, they should use objective outcome measures and have long follow-up periods. This approach will help us better understand how long treatments work and how safe they are over time.

To improve treatments for ASD, a comprehensive approach is necessary. This approach should include genetic factors, gene regulatory mechanisms, the workings of neural circuits, and environmental influences. Genome-wide association studies and transcriptomic analyses have pinpointed many genes related to ASD, specifically those involved in GABA synthesis, receptor assembly, and synaptic regulation (Gogate et al., 2024). Additionally, epigenetic mechanisms like promoter methylation of GABA receptor subunit genes add complexity to the regulation of inhibitory pathways (Herrera et al., 2024). Emerging technologies strongly support translational research. Using patient-derived induced pluripotent stem cells and brain organoids allows for detailed modeling of individual neurodevelopmental processes and how they respond to drugs.

Using high-throughput screening to identify specific modulators for different subtypes can accelerate the identification of therapeutically effective compounds. Moreover, systems biology approaches incorporating machine learning can assist in detecting vulnerabilities within biological networks and support the development of tailored treatments (Hali et al., 2025).

Upcoming interventions for ASD are expected to use targeted neurotherapeutic methods, employing substances that selectively bind to specific receptor subtypes. These treatments will be administered during critical developmental periods and guided by validated biological markers. This method seeks to improve key behavioral symptoms, restore neural circuit function, and minimize side effects. Additionally, combining systems biology approaches with machine learning can help identify weaknesses in biological networks and facilitate the creation of personalized therapies (Hali et al., 2025).

## 9 Conclusion

Research across genetics, molecular biology, electrophysiology, and clinical fields highlights the crucial role of GABA_A_ receptor dysfunction in ASD. Alterations in receptor subunit composition, reduced GABA production, and chloride homeostasis problems disrupt inhibitory signaling, leading to E/I imbalance, a key neurobiological feature of ASD. Nonetheless, challenges such as subunit specificity, developmental timing, and the absence of reliable biomarkers for patient selection persist. Future efforts should focus on personalized neurotherapies by combining genetic testing, advanced imaging, and innovative models to customize GABA-based treatments for each person’s neurodevelopmental profile. These approaches can turn mechanistic insights into targeted, effective therapies for ASD. Future research should prioritize precision neurotherapeutic strategies that integrate genetic profiling, multimodal neuroimaging, and receptor subtype-specific pharmacology to restore inhibitory circuit function in ASD.
